# 基于非靶向代谢组学探索辐射诱导的小鼠肺上皮细胞代谢改变

**DOI:** 10.3779/j.issn.1009-3419.2024.106.28

**Published:** 2024-10-20

**Authors:** Hao FAN, Xiangwei GE, Xin ZHOU, Yao LI, Qiaowei LIU, Yi HU

**Affiliations:** ^1^100853 北京，中国人民解放军医学院; ^1^Chinese PLA Medical School, Beijing 100853, China; ^2^100071 北京中国人民解放军总医院第五医学中心肿瘤医学部; ^2^Department of Oncology, the Fifth Medical Center, Chinese PLA General Hospital, Beijing 100071, China

**Keywords:** 辐射, 代谢组学, MLE12细胞, 核苷酸代谢, Radiation, Metabolomics, MLE12 cells, Nucleotide metabolism

## Abstract

**背景与目的:**

代谢变化是放射性肺炎的重要特征之一。放疗作为胸部肿瘤治疗的常规手段，在有效杀伤肿瘤细胞的同时也会引发局部炎症和纤维化等不良反应，进而导致治疗效果受限，严重影响患者生活质量。因此，深入探讨放疗引起的代谢改变具有重要意义。本研究旨在探究X射线辐射对小鼠肺上皮细胞系（murine lung epithelial-12, MLE12）代谢的影响。

**方法:**

于体外环境下培养MLE12细胞系，并将其随机分为辐射组（radiation group，IR组）与对照组（control group，NC组）。使用日立X射线辐照仪以10 Gy的剂量对IR组细胞进行辐射处理。辐射后48 h收集细胞上清液样本。通过液相色谱-质谱联用仪（liquid chromatograph mass spectrometer, LC/MS）对样本进行代谢组学分析。

**结果:**

LC/MS代谢组学分析揭示了MLE12细胞在辐射后48 h的代谢变化。与NC组相比，IR组中共有38种分泌型代谢物发生改变。根据京都基因与基因组百科全书（Kyoto Encyclopedia of Genes and Genomes, KEGG）数据库的注释，差异代谢物主要涉及核苷酸代谢、氨基酸代谢和脂质代谢等通路，其中核苷酸代谢的差异最为显著。

**结论:**

X射线辐射对MLE12细胞的代谢产生了显著影响，主要影响核苷酸代谢途径，包括嘌呤和嘧啶等代谢产物及相关代谢通路。

## Abstract (en)

**Background and objective:**

Metabolic change is one of the important characteristics of radiation pneumonitis. Radiotherapy, as a conventional method for the treatment of thoracic tumors, can not only effectively kill tumor cells, but also cause adverse reactions such as local inflammation and fibrosis, which leads to limited therapeutic effect and profound impact on the quality of life of patients. Therefore, it is of great significance to explore the metabolic changes caused by radiotherapy. The aim of this study was to investigate the effects of X-ray irradiation on the metabolism of a mouse lung epithelial cell line (murine lung epithelial-12, MLE12).

**Methods:**

MLE12 cells were cultured in vitro and randomly divided into radiation group (IR) and control group (NC). Cells in the IR group were irradiated at a dose of 10 Gy using a Hitachi X-ray irradiator. Cell supernatant samples were collected at 48 h after irradiation. Metabolomic analysis of the samples was performed by liquid chromatograph mass spectrometer (LC/MS).

**Results:**

LC/MS metabolomics analysis revealed the metabolic changes of MLE12 cells at 48 h after irradiation. A total of 38 secretory metabolites were altered in the IR group compared with the NC group. According to the annotation of Kyoto Encyclopedia of Genes and Genomes (KEGG) database, the differential metabolites are mainly involved in nucleotide metabolism, amino acid metabolism and lipid metabolism, among which the difference in nucleotide metabolism is the most significant.

**Conclusion:**

The metabolism of MLE12 cells was significantly affected by X-ray irradiation, mainly affecting the nucleotide metabolic pathways, including purine and pyrimidine metabolites and related metabolic pathways.

放疗是癌症治疗不可或缺的一环，惠及超过半数接受综合治疗的癌症患者^[1]^。通过高能粒子束破坏肿瘤细胞的DNA，放疗能够有效遏制肿瘤细胞的分裂与复制，进而缩小甚至清除肿瘤。然而，放疗在展现其强大疗效的同时，也会引发正常细胞损伤并释放炎性因子，导致局部炎症及纤维化等不良反应，这些副作用不仅限制了治疗效果，还严重影响了患者的生活质量，给患者长期生存带来困扰^[2]^。

细胞微环境变化是细胞生存、代谢改变的重要原因，异常代谢环境会导致不同疾病的发生。研究^[3⇓-5]^表明，放疗可导致一系列细胞微环境变化，直接或间接诱导细胞DNA损伤，引起肿瘤细胞和肺上皮细胞的衰老和凋亡，也可通过损伤线粒体、诱发细胞内氧化还原反应紊乱、激活炎性细胞等方式，进一步加剧炎症反应的发生，然而究竟是何种物质引起了免疫细胞激活目前仍不清楚。有研究^[6,7]^表明放疗后上皮细胞释放出细胞因子激活免疫细胞，最终引发炎症反应。随着代谢组学的不断发展，普遍认为电离辐射引起肺上皮细胞损伤会影响细胞代谢，继而影响免疫细胞的微环境，造成不同的病理生理反应。

代谢组学通过对分子量低于1000的代谢产物的精准定量，能够深刻揭示生物体的生理及病理状态，是细胞下游表型的分析形式。细胞上清液作为体外细胞微环境的核心成分，可以直接映射出体外模型的微环境特征^[8,9]^。近年来代谢组学在辐射后小鼠模型及放疗后人体肺组织的研究中得到了广泛应用，但这些研究^[10⇓-12]^大多聚焦于宏观样本，如血清、尿液等，目前国内外尚未见有关肺上皮细胞体外辐射模型的相关报道。本研究旨在以小鼠肺上皮细胞（murine lung epithelial-12, MLE12）为基础，构建相对稳定的体外辐射模型，用于分析辐射对肺上皮细胞代谢的影响；并使用代谢组学的研究和分析方法，识别辐射后参与炎症反应调控的关键代谢物质及相关代谢途径。这不仅有利于理解放疗导致不良反应的发生机制，更为优化放疗策略、减轻患者负担提供了科学依据。

## 1 材料与方法

### 1.1 主要材料及分析仪器

#### 1.1.1 实验细胞及试剂

MLE12细胞株（北纳生物细胞服务资源平台）；DMEM-H/F12培养基（C11330500BT, Gibco）；胎牛血清（Cat:ST30-3302, PAN seratech）；青霉素/链霉素（Solarbio, Cat:P7630）；彗星实验试剂盒（C2041M，碧云天生物技术公司）；CCK-8（Cell Counting Kit-8）试剂盒（40203ES60，翌圣生物科技公司）；RIPA裂解液（G2002）、BCA试剂盒（G2026）、蛋白酶抑制剂（G2006、G2007、G2008）、SDS-PAGE上样缓冲液（G2075）、TBST溶液（G0004）、ECL试剂（G2014）购自武汉赛维尔生物科技有限公司；部分抗体γ-H2AX（#9718）、NRF2（#12721）购自美国CST公司；P16（ab108349）和P21（ab109520）购自英国Abcam公司；α-Tublin（11224-1-AP）、p53（60283-2-Ig）、GAPDH（60004-1-Ig）购自武汉三鹰生物技术公司。

#### 1.1.2 主要实验设备

质谱仪（Q Exactive HF-X）、色谱仪（Ulimate 3000）、低温离心机（Heraeus Fresco 21）购自美国Thermo公司；色谱柱（HSS T3）购自美国Waters公司；X射线辐照仪（MBR-1520R-3）购自日本日立解决方案（中国）有限公司。

### 1.2 实验方法

#### 1.2.1 细胞培养

MLE12细胞在DMEM-H/F12培养基中培养，附加2%胎牛血清和1%青霉素/链霉素，细胞培养在37 ^o^C的5% CO_2_孵育箱中进行，当细胞密度达到80%-90%时进行传代或X射线辐射。

#### 1.2.2 样品采集方案

在6孔板中每孔铺板1×10^6^个MLE12细胞，约24 h后密度达80%-90%，更换新鲜完全培养基后进行辐射，总剂量为10 Gy，辐射剂量率在（1.4±0.1）Gy/min，辐射完毕后在细胞孵箱中继续培养48 h，吸取细胞上清液至少1 mL，离心（14,000 g, 4 ^o^C, 10 min），吸取上清液，液氮速冻15 min后-80 ^o^C冰箱保存，运输及后续实验过程全程足量干冰保温，辐射组（radiation group，IR组）与对照组（control group，NC组）均使用6个重复的生物学样本。

#### 1.2.3 样品处理及制备方案

取100 μL原始样本，加入500 μL -80 ℃预冷的甲醇，涡旋振荡1 min；于4^ o^C静置10 min；4^ o^C，14,000 g离心20 min；取出200 μL上清液后，低温冻干，保存于-20 ℃冰箱。从每个实验样本中取等体积样本混匀作为质量控制（quality control, QC）样本。空白对照样本为20%甲醇，并采用与实际样品同样的处理流程和数据采集方法。进样前先进行QC样本的检测，之后在样本检测中间插入QC样本，用于评估整个实验过程中平台稳定性。

#### 1.2.4 彗星实验方案

取对数期生长的MLE12细胞，以不同剂量（0、2、4、6、8、10 Gy）方案进行辐射，辐射后立即收集包括上清液在内的所有MLE12细胞，使用彗星实验试剂盒（C2041M，碧云天生物技术公司）进行实验。

#### 1.2.5 Western blot实验方案

将对数期生长的MLE12细胞辐射10 Gy，1 h后收集6孔板中IR组与NC组的细胞，用磷酸盐缓冲液清洗3次，使用RIPA裂解液提取细胞总蛋白并使用BCA（bicinchoninic acid assay）法测定蛋白浓度，裂解完毕后按比例加入SDS-PAGE上样缓冲液，100^ o^C水浴变性15 min，制备合适浓度的聚丙烯酰胺胶，取20 μg蛋白上样电泳，转膜采用湿转法转膜，5%脱脂牛奶封闭1 h，使用TBST（Tris Buffered Saline with Tween-20）溶液按照1:1000的比例稀释一抗，4^ o^C摇床孵育过夜，TBST洗膜3次，使用TBST溶液按照1:10,000的比例稀释二抗，室温孵育1 h，TBST洗膜3次，使用ECL试剂显影。

#### 1.2.6 CCK-8测定细胞增殖活力方案

用培养基将IR组（10 Gy）与NC组（0 Gy）的细胞密度调整为1×10^4^个/mL，每孔100 μL细胞悬液接种于96孔板，在细胞孵箱中培养24-48 h。加入CCK-8试剂10 μL，在细胞培养箱中孵育1.5 h，应用酶标仪在波长450 nm处测量每孔吸光度（optical density, OD）值。

### 1.3 统计学方法

实验数据使用Graphpad Prism 9.5版本软件进行统计分析，计量资料包括彗星实验尾长、蛋白相对表达水平等，两组之间差异比较采用t检验，多组间差异比较采用方差分析，P<0.05为差异具有统计学意义。

## 2 结果

### 2.1 辐射对MLE12细胞影响

按照上述方法使用X射线辐照仪对MLE12细胞进行不同剂量辐射后，设计彗星实验验证细胞损伤程度。在荧光显微镜下观察，辐射剂量越高，MLE12细胞拖尾效应越明显，提示高剂量的X射线对MLE12细胞DNA的损伤更明显。使用图像分析软件Image J测量细胞尾长，每个剂量组随机选取5个细胞测量尾长，得到结果如图1A所示，在10 Gy的辐射剂量下MLE12细胞损伤最明显，与其他剂量组相比，差异具有显著的统计学意义（P<0.05）。Western blot实验结果显示，辐射后的MLE12细胞中，衰老标志物p16、p21蛋白，损伤应激标记物γ-H2AX、NRF2蛋白，以及基因修复标记物p53蛋白均出现明显的升高（图1B），提示10 Gy辐射后MLE12细胞发生了损伤、衰老、应激反应及修复基因的启动，证明体外实验中10 Gy的辐射剂量对细胞产生显著影响。随后利用CCK-8细胞增殖实验检测MLE12细胞在辐射后24和48 h的增殖活力。实验结果（图1C）表明，辐射后24 h细胞增殖活力并未受到明显影响，而辐射后48 h细胞增殖活力出现显著变化。由于细胞的增殖活力与代谢有重要关系^[13,14]^，推测MLE12细胞代谢受到辐射时间的影响，在辐射后24 h代谢水平未发生明显改变，而辐射后48 h可能出现代谢改变，因此选择10 Gy辐射后48 h的MLE12细胞上清液作为代谢组检测样本，进一步揭示辐射对细胞代谢的具体影响。

**图1:**
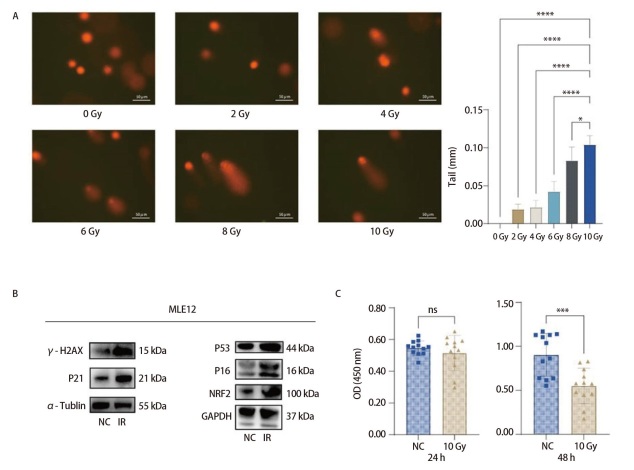
辐射对MLE12细胞影响。A：彗星实验示不同剂量辐射对MLE12细胞DNA损伤；B：Western blot检测MLE12细胞辐射后衰老、损伤、应激及修复相关标志物；C：辐射后不同时间MLE12细胞增殖活性。*P<0.05; ***P<0.001; ****P<0.0001。

### 2.2 X射线辐照仪诱导MLE12细胞的非靶向代谢组学分析

代谢组学能够精准揭示代谢物丰度差异并富集关键代谢通路，本研究希望着重分析高剂量辐射对MLE12细胞的代谢改变，以及辐射后细胞微环境发生变化的情况，根据上述实验结果设计代谢组学实验，最终流程见图2。由于从代谢组中获取的数据相对复杂，我们采用了QC样本法对最终获取的定量数据结果进行归一化处理，以确保分析的准确性。在阴离子模式下共鉴定并注释到508种代谢物，阳离子模式下共鉴定并注释到482种代谢物。

**图2:**
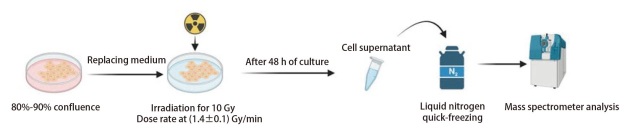
细胞辐射及上清液收集过程示意图

### 2.3 主成分分析

采用主成分分析的策略对数据进行降维，力求通过取出少数的综合变量能最大限度地反映原始数据的综合信息，主成分分析二维图可以看到QC样本相互靠近且聚集，提示仪器检测稳定性、重复性良好，在阴离子模式下仅有NC组的两个样品落入了IR组的置信区间内，而阳离子模式下不同样本组之间的良好聚集见图3，提示辐射对小鼠MLE12细胞系的代谢明显产生影响，代谢物彼此分离，提示整体分组合理，样本质控合格。

**图3:**
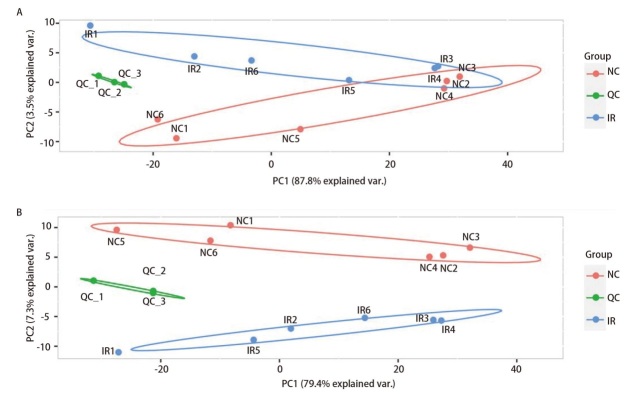
主成分分析。A：阴离子模式；B：阳离子模式。

### 2.4 差异代谢物筛选

运用多元统计的方法对采集的多维数据进行降维和回归处理，以便后续筛选分析差异代谢物。将两种模式下代谢物定量值归一化后按照差异倍数（fold change, FC）与变量投影重要度（variable importance projection, VIP）值对所有代谢物进行筛选，设置阈值为VIP>1.0，差异倍数FC>1.5或FC<0.667且P<0.05，共筛选出38种差异代谢物，阴离子模式下有8种代谢物在辐射后上调，无下调代谢物；阳离子模式下有20种代谢物在辐射后上调，10种代谢物下调，其中包含1个模式内重复代谢物，上述所有差异代谢物根据人类代谢数据库（Human Metabolome Database, HMDB）注释去除重复及无意义值，共得到有注释的差异代谢物21种，按t检验后P值差异依次排列见表1，这21种差异代谢物包括核苷及其衍生物、短肽、氨基酸及其衍生物、芳香族化合物、脂质等。有两种代谢物（HMDB0000273、HMDB0000292）在两种模式下都有差异，均表现为辐射后上调。通常两种离子模式均显示出电离差异的代谢物差异性最为显著，也最具代表性。

**表1:** MLE12细胞辐照组对比对照组差异代谢物

HMDB_ID	Name	VIP	FC (IR_VS_NC)	P	Signature	Nucleotides and their analogs
HMDB0000292	Xanthine	2.9363	2.7121	6.61E-10	Up	√
HMDB0000157	Hypoxanthine	4.3954	4.4530	6.69E-09	Up	√
HMDB0000630	Cytosine	4.3556	4.4026	1.18E-08	Up	√
HMDB0000132	Guanine	4.584	4.7734	3.28E-08	Up	√
HMDB0000273	Thymidine	11.429	45.6846	1.07E-07	Up	√
HMDB0011172	γ-Glutamyl-L-valine	5.1014	0.1779	6.27E-07	Down	
HMDB0011171	γ-Glutamyl-L-leucine	4.7246	0.2031	2.54E-06	Down	
HMDB0031768	Iprobenfos	5.5834	49.1349	3.20E-06	Up	
HMDB0000195	Inosine	3.6819	3.4355	8.08E-06	Up	
HMDB0000089	Cytidine	4.1481	4.0612	8.45E-06	Up	√
HMDB0039002	Mytilin A	4.4503	25.4397	1.81E-05	Up	
HMDB0000012	Deoxyuridine	2.7886	7.2548	3.57E-05	Up	√
HMDB0004610	Phytosphingosine	5.1016	0.1900	5.71E-05	Down	
HMDB0030505	Bargustanine	2.3979	2.3057	3.31E-03	Up	
HMDB0259840	Visnagin	1.5531	0.5764	5.08E-03	Down	
HMDB0001069	2-phenylaminoadenosine	2.8931	16.1546	5.48E-03	Up	√
HMDB0011152	LysoPE (P-16:0/0:0)	1.7928	1.8692	6.54E-03	Up	
HMDB0003333	8-hydroxy-deoxyguanosine	2.4848	2.2083	8.63E-03	Up	√
HMDB0248792	Azelnidipine	2.3765	0.4333	8.99E-03	Down	
HMDB0000759	Glycyl-L-leucine	1.6926	0.5627	1.61E-02	Down	
HMDB0014677	Ciprofloxacin	1.5944	1.6755	2.01E-02	Up	

HMDB_ID: Human Metabolome Database Identity Document; VIP: variable importance projection; FC: fold change.

### 2.5 差异代谢物判别

为了区分两两分组间的代谢谱差异，采用正交偏最小二乘法判别分析（orthogonal partial least squares discriminant analysis, OPLS-DA）对模型作严格的验证。观察打乱前的原始模型在200次随机打乱样本的随机置换检验（permutation test），分别计算各自打乱后模型的R2Y和Q2，绘制置换检验图（图4），无论是阴离子模式还是阳离子模式下模型可解释变量R2Y相关性系数均接近1，提示差异代谢物的拟合效果好，模型Q2>0.4，提示差异代谢物模型可预测度较高。

**图4:**
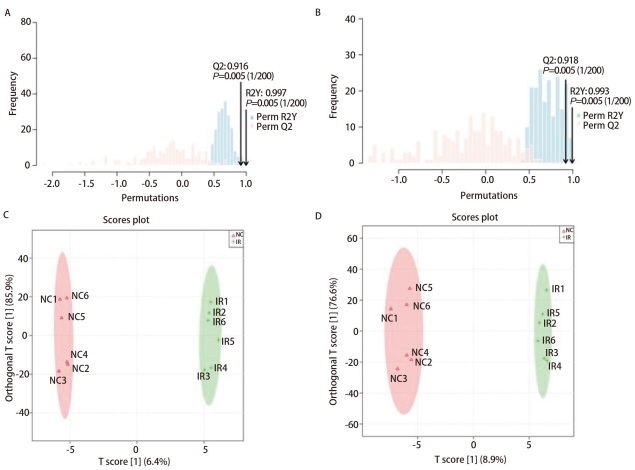
OPLS-DA置换检测与OPLS-DA分析。A：阴离子模式下OPLS-DA置换检测；B：阳离子模式下OPLS-DA置换检测；C：阴离子模式下OPLS-DA分析；D：阳离子模式下OPLS-DA分析。

### 2.6 差异代谢物分析

鉴于本研究核心在于辐射后导致细胞微环境的变化，因此深入分析了所有的差异代谢物参与的代谢通路，并与京都基因与基因组百科全书（Kyoto Encyclopedia of Genes and Genomes, KEGG）数据库进行注释比对，找出与辐射相关性最高的代谢通路，进行KEGG通路富集分析。结果显示嘌呤代谢与嘧啶代谢在KEGG通路富集气泡图中富集均较为显著，两种模式下均排名前三位（图5），提示辐射对MLE12细胞核苷酸代谢相关通路影响最为明显。

**图5:**
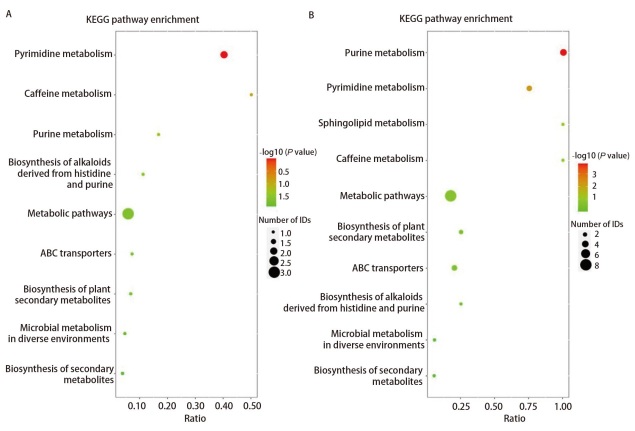
KEGG通路富集分析气泡图。A：阴离子模式；B：阳离子模式。

## 3 讨论

肺上皮细胞作为肺泡的主体组成部分，具有抵御病原体、支持呼吸功能、参与免疫反应等重要作用，其直接暴露于大气中各类成分，是人体中最容易受到损伤的部位之一。胸部肿瘤患者在接受放疗后肺上皮细胞遭到严重破坏，其代谢变化势必会影响其他细胞功能，以往研究^[15,16]^发现辐射可以对细胞微环境产生明显影响，在短期内诱发炎症反应，这可能与肺上皮细胞代谢改变具有密切关系。本研究聚焦于高能射线引起肺上皮细胞发生代谢的改变，模拟胸部肿瘤患者接受放疗的总剂量、剂量率等关键治疗条件，以此来反映生物体放疗后的细胞微环境状态。通过代谢组学方法定量分析代谢产物，借助公共数据库探索其中代谢产物的变化，揭示肺上皮细胞在辐射过程中受损伤产生的特殊代谢物对细胞微环境影响的具体机制。

本研究结果显示，MLE12细胞在10 Gy剂量的X射线辐射后，出现了明显的DNA损伤及细胞衰老，并激活了细胞修复相关基因，在辐射后48 h细胞增殖活力出现明显下降，因此，研究团队认为经过10 Gy剂量辐射后48 h，MLE12细胞的周期受到影响，细胞代谢可能发生改变。进一步对辐射后48 h的MLE12细胞上清液进行代谢组学分析，发现核苷及其衍生物在差异代谢物中占比最高，约为42.9%（第1、2、3、4、5、10、12、16、18种物质），且在两种模式下均有差异的2种物质（HMDB0000273、HMDB0000292）均为核苷及其衍生物，强烈提示辐射对肺上皮细胞核苷酸代谢产生了显著影响。同时，辐射对氨基酸、糖类和脂类代谢也产生了一定的影响。

电离辐射作用于生物大分子，导致分子的电离和激发，从而引起生物大分子和生物膜的损伤，这是辐射生物效应的基础^[17⇓-19]^。在本研究中电离辐射引起核苷酸代谢的改变具体表现为细胞微环境中核苷及其衍生物含量上升，而国内外研究^[20⇓-22]^表明，核苷酸摄入量异常会导致免疫功能改变，赵明等^[23]^的研究结果显示缺乏核苷酸喂养的小鼠淋巴细胞增殖能力较正常小鼠下降，免疫功能受损；Chandra等^[24]^认为喂食缺乏核苷饮食的动物淋巴细胞对有丝分裂原的刺激反应减少，白介素-2的产生受损，但在食物中添加过量的核苷酸并不会提高或激活免疫功能，这可能是由于食物中过量的核苷酸无法直接提高细胞微环境中的核苷酸含量所致。肺癌患者经过放疗后往往会出现放射性肺炎、肺纤维化等一系列不良反应，这些局部炎症反应与免疫细胞激活密切相关，因此，本研究推测，辐射导致的细胞微环境中核苷酸含量上升及相关代谢通路发生改变是免疫细胞激活的重要原因。

DNA断裂损伤是电离辐射引起的最严重损伤之一，可以引发一系列DNA损伤反应^[17]^，放疗患者常面临短期或长期的辐射后损伤风险，这些不良反应的严重程度与受辐射的剂量直接相关^[25]^。目前，关于辐射引起代谢变化的研究主要还集中在糖类和脂质代谢上，在基础研究方面，Amorim等^[26]^的研究证实辐射会导致小鼠糖类摄取减少、脂肪酸合成受损；Bacarella等^[27]^认为对恒河猴的全身辐射会导致代谢性疾病，造成胰岛素抵抗型糖尿病的发生；Laiakis等^[28]^关于辐射后血清脂质组学的分析指出辐射可能是导致高脂血症和炎症反应的重要原因。临床研究方面，Arenas等^[29]^对比了乳腺癌患者放疗前后血清代谢产物的变化，结果显示胸部单纯放疗对糖酵解、三羧酸循环、氨基酸代谢均有较大变化；Wang等^[30]^对血清相关代谢产物的分析指出晚期直肠癌患者接受新辅助放化疗后氨基酸代谢通路受到了显著影响。本研究重点关注体外模型的构建，发现核苷酸代谢改变是辐射引起肺上皮细胞微环境变化的主要因素。目前已有多种局部注射核苷与寡核苷酸类药物疗法应用于临床试验和临床治疗，主要针对代谢疾病、血液疾病等^[31⇓⇓-34]^，含有特定序列的寡核苷酸可以改变免疫条件，提高免疫刺激作用，与免疫治疗或放疗联合可能具备临床价值^[35⇓-37]^，而参与DNA修复或氧化应激反应的蛋白质在基因层面的单核苷酸多态性（single nucleotide polymorphisms, SNPs）可能与放疗的远期不良反应密切相关^[38]^，虽然局部的免疫激活可能导致一些不良反应的发生，但不可否认的是，由核苷酸含量改变导致免疫激活的治疗方法可能在临床中具有广阔的治疗前景。

体内细胞微环境复杂，以细胞上清液反映细胞微环境虽然具有一定的局限性，但是能够在控制单一变量的情况下对上皮细胞的改变进行评估，更有利于从简到繁解析复杂微环境的特点，破译体内微环境下的细胞互作通讯。使用细胞培养上清液反映细胞微环境也是国内外常用的研究方法，如使用细胞上清液对其他细胞进行刺激以研究细胞间通讯、提取体外培养细胞的外泌体用于治疗等^[39⇓-41]^。此外，受限于体外模型，本研究无法探索临床中常用的小剂量分割治疗方案对肺上皮细胞微环境产生的影响（如2 Gy连续辐射10次）。在剂量选择方面参考了国内外体外模型的常规剂量^[3,42,43]^，最终决定采用临床常用的最高治疗剂量10 Gy进行实验，而未能探索更高剂量（可能源自新型放疗设备、核武器暴露或职业危害）对肺上皮细胞代谢的潜在影响。本研究结论认为，辐射导致MLE12细胞核苷酸代谢改变。而代谢变化可能影响免疫激活，进一步导致免疫不良反应的发生^[15,16]^。未来的研究可以通过构建更为复杂的体内模型，例如屏蔽小鼠头腹部而仅对胸部进行辐射，模拟放射性肺炎的病理过程，进一步检测体内模型中核苷酸含量的变化，确立免疫功能激活与核苷酸代谢之间的内在联系。本研究围绕辐射对肺上皮细胞的代谢改变展开的初步探索为深入研究放射性肺损伤的代谢调控及靶向干预奠定了基础。
